# DoE-oriented optimization of a chitosan-based nanoemulgel of clobetasol propionate for therapeutic intervention in psoriasis

**DOI:** 10.1039/d6ra04200g

**Published:** 2026-08-04

**Authors:** Shiv Kumar Prajapati, Deepa Lashkari, Sweta Acharya, Meenakshi Bajpai, Ankit Jain

**Affiliations:** a Institute of Pharmaceutical Research, GLA University NH-2, Mathura-Delhi Road Mathura UP India; b Department of Pharmacy, Birla Institute of Technology and Science-Pilani Pilani Campus Rajasthan 333031 India ankit.j@bits.pilani-bits.ac.in

## Abstract

Psoriasis is an inflammatory skin disease caused by immune-mediated inflammation. Clobetasol propionate (Cp), a steroid, reduces inflammation by binding the glucocorticoid receptor. This study aimed to optimize a Cp-loaded nanoemulsion (Cp-NE) with a focus on its cytocompatibility with HaCaT keratinocytes and physicochemical stability during storage. Optimization was performed using a Box–Behnken design. Cp-NE was loaded into a chitosan gel to prolong skin retention. The formulations were analyzed for globule size, PDI, ZP, and viscosity. *In vitro*, drug release and skin permeability were also assessed. The optimized Cp-NE had a globule size of 122 nm, a %EE of 89%, a PDI of 0.508, and a ZP of −28 mV. The Cp-NE gel had a pH of 6.13 and a viscosity of 6799 cps, making it suitable for topical use. *In vitro*, free Cp released 91% within 2 h; Cp-NE released 83.4% at 12 h, indicating sustained release, which was further extended in chitosan gel. The release followed Higuchi kinetics, confirming diffusion control. Skin permeation was higher with the Cp-NE gel than with the free drug. Cytocompatibility tests showed higher cell viability for Cp-NE. Cytokine assays in HaCaT cells showed significant reductions in IL-6 and TNF-α with Cp-NE and Cp-NE gel, indicating anti-inflammatory potential. MTT assays confirmed biocompatibility, supporting transdermal use. The results suggest that chitosan nanoemulsions improve drug delivery and biocompatibility and reduce inflammation. Stability studies showed excellent stability at 5 ± 3 °C and 25 ± 2 °C/60 ± 5% RH. The Cp-NE gel exhibited favorable physicochemical properties, sustained release, enhanced skin permeation, and stability, highlighting its potential for psoriasis treatment.

## Abstract 2

Psoriasis is an inflammatory skin disease caused by immune-mediated inflammation.

## Graphical abstract

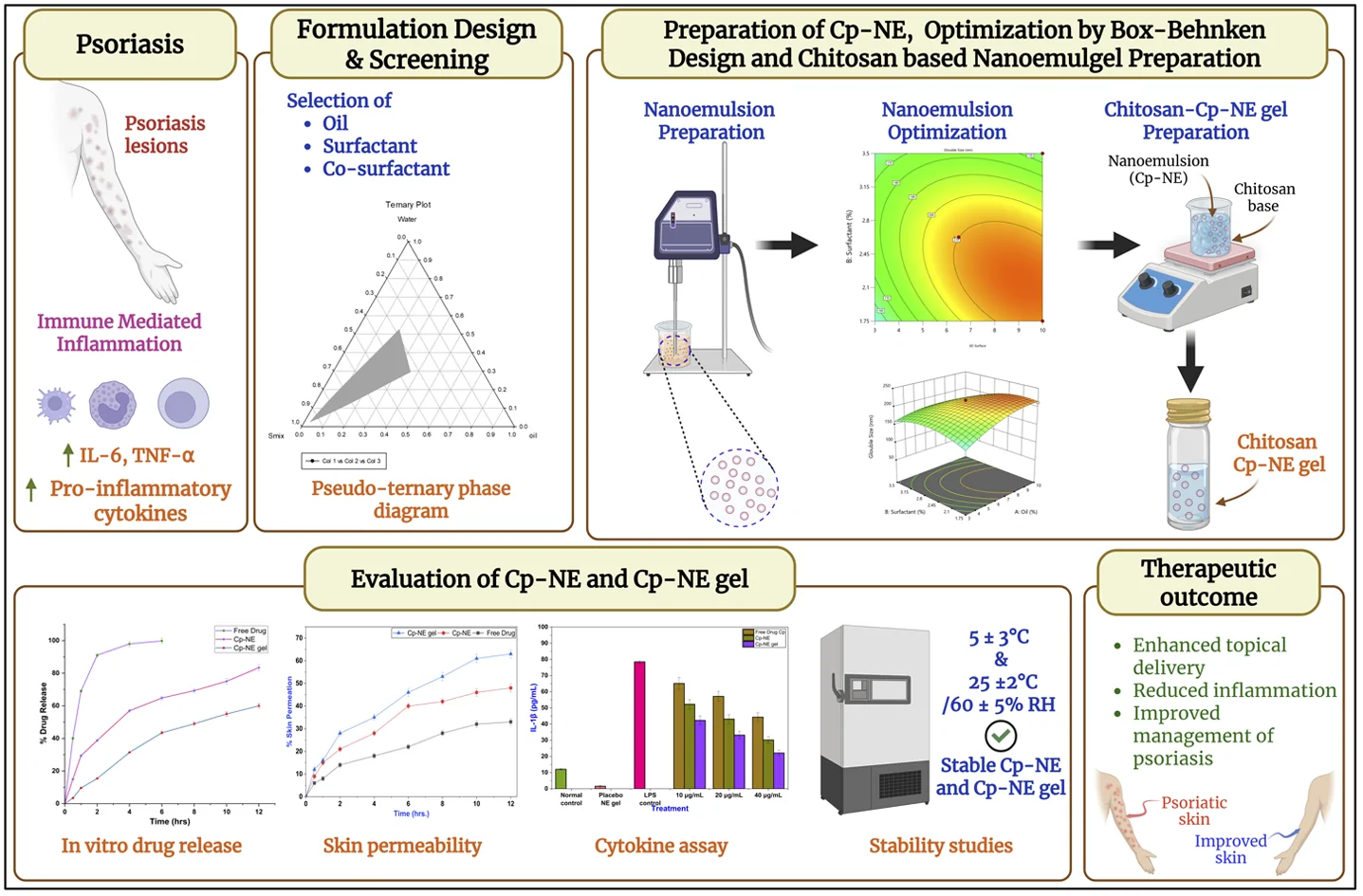

## 1. Introduction

“Psoriasis is a chronic inflammatory skin disease” triggered by a dysregulated immune response, with both genetic and environmental factors.^1,2^ Worldwide, psoriasis affects over 100 million people, with a global prevalence of 1.12%, ranging from 0.38% to 2.84% across geographic and demographic factors. The pathogenesis of psoriasis is characterized by keratinocyte proliferation and the infiltration of inflammatory immune cells into the epidermis and dermis. The above symptoms are primarily attributed to immune signaling dysfunction, including the IL-23/Th17 axis, which induces chronic inflammation and disrupts the skin's homeostatic balance.^3^ Psoriasis can affect joints in about one-third of cases, leading to psoriatic arthritis. It is linked to comorbid conditions such as psychological illnesses and cardiovascular disorders.^4^ Conventional topical treatment approaches are considered the first-line management for psoriasis. However, their efficacy is impeded by poor skin penetration, ineffective drug delivery, and potential toxic side effects at higher doses.^5,6^ Topical therapies remain the first-line treatment for mild-to-moderate psoriasis and include corticosteroids (clobetasol propionate, betamethasone dipropionate),^7^ vitamin D analogues (calcipotriol),^8^ retinoids (tazarotene),^9^ calcineurin inhibitors (tacrolimus and pimecrolimus),^10,11^ and keratolytic agents such as salicylic acid.^12^ Systemic therapies include methotrexate, cyclosporine, acitretin, apremilast, and biologics targeting TNF-α, IL-17, and IL-23 pathways.^13^ However, all the above treatment modalities lack safety, efficacy, and curability. Topical administration is the most popular due to its low toxicity and minimal systemic uptake. Still, there is poor skin penetration, particularly in the thickened psoriatic skin.^14^ Clobetasol propionate (Cp) is a potent topical glucocorticoid with pronounced anti-inflammatory, antipruritic, vasoconstrictive, and immunomodulating attributes. This drug is widely used to treat skin diseases characterized by excessive proliferation and inflammation, such as psoriasis and atopic dermatitis, and has demonstrated high effectiveness and safety. The pharmacological effect is achieved by stimulating the synthesis of phospholipase A_2_ inhibitors, thereby inhibiting the secretion of arachidonic acid and the production of inflammatory substances. Although the compound exhibits compelling therapeutic activity as a first-class corticosteroid, prolonged use may cause skin atrophy, telangiectasia, striae, irritation, and, in severe cases, hypothalamic–pituitary–adrenal axis suppression.^15,16^ For this, a nanoemulsion (NE) formulation is developed in this study. NE is an aqueous oil phase stabilized with an emulsifier to increase the solubility of hydrophobic drugs in the form of nanoparticles, which increases the thermodynamic activity and skin penetration.^17,18^ The NE is stable with respect to dispersion and optical clarity, making it a good choice for a delivery system in psoriasis treatment. Its function in enhancing solubility is achieved by forming nanoparticles that contain poorly soluble drugs. Water removes dead skin cells and scales, while the nanoparticles increase thermodynamic activity, facilitating deeper skin penetration.^19^ Chitosan is a positively charged polymer, water- and oxygen-permeable, with hemostatic properties and antimicrobial activity for treating skin infections.^20,21^ With properties such as biocompatibility, biodegradability, and the ability to enhance drug permeation, it can be used as a suitable polymer to control the delivery rate.^22,23^ This study focused on optimizing Cp-NE for topical delivery using a BBD experimental design. To enhance skin retention and improve percutaneous permeation efficiency, the optimized Cp-NE formulation was further loaded into a chitosan gel matrix.

## 2. Materials and methods

Cp was obtained from Praveen Chemicals India Pvt. Ltd; propylene glycol, PEG-400, and Tween were purchased from CDH Fine Chemicals; and Chitosan was obtained from Sigma-Aldrich (Milan, Italy). The other reagents/ingredients used in this research were of analytical grade. HaCaT cells and RAW264.7 murine macrophages were obtained from Professor Gautam Singhvi's laboratory at the Birla Institute of Technology and Science, Pilani, Pilani Campus, Rajasthan – 333031.

### 2.1. Screening of formulation variables

#### 2.1.1. Selection of oils

The solubility of Cp in lipid(s) such as oleic acid, olive, peanut, sunflower, castor, and coconut oil was assessed by adding an excess of the drug to 2 mL of each lipid phase. Mixtures were stirred and shaken to reach equilibrium, centrifuged at 3000 rpm for 15 min, and filtered through a 0.22 µm membrane. Cp concentration was measured by preparing ethanol dilutions and recording the absorbance at 254 nm using UV-visible spectroscopy. Oleic acid was selected as the oil component due to its high solubility in clobetasol propionate and its proven ability to enhance skin penetration. The selection of Tween-80 as a surfactant was based on its non-ionicity, high HLB value (≈15), and its ability to stabilize oil-in-water NE. Moreover, PEG-400 was taken as a co-surfactant owing to its ability to reduce interfacial tension and increase interfacial film flexibility.^24^

#### 2.1.2. Selection of surfactants

Surfactants, including Tween-80, Tween-20, Tween-60, Tween-40, and IPM, were tested in NE systems. An aqueous surfactant solution (15% w/w, 2.5 mL) was prepared, and 2 mL of oil was added with vigorous mixing, yielding a transparent, one-phase solution. The oil phase was then added gradually until the formulation was achieved.^25^ Hydrophilic surfactants are used in this because the target formulation is an oil-in-water NE. High HLB values surfactants present enhanced emulsification efficiency, improved globule stabilization, and greater long-term stability of the dispersed system.^26,27^

#### 2.1.3. Selection of co-surfactants

Tween-80 was combined with cosurfactants, including ethanol, isopropyl alcohol, PEG 400, and PG, to prepare the *S*_mix_ at a 1 : 1 ratio for phase diagram construction. Formulations with oil-to-*S*_mix_ ratios from 9 : 1 to 1 : 9 mapped phase boundaries and identified the NE region across a broad range.

### 2.2. Method of preparation for Cp-NE

**Fig. 1:**
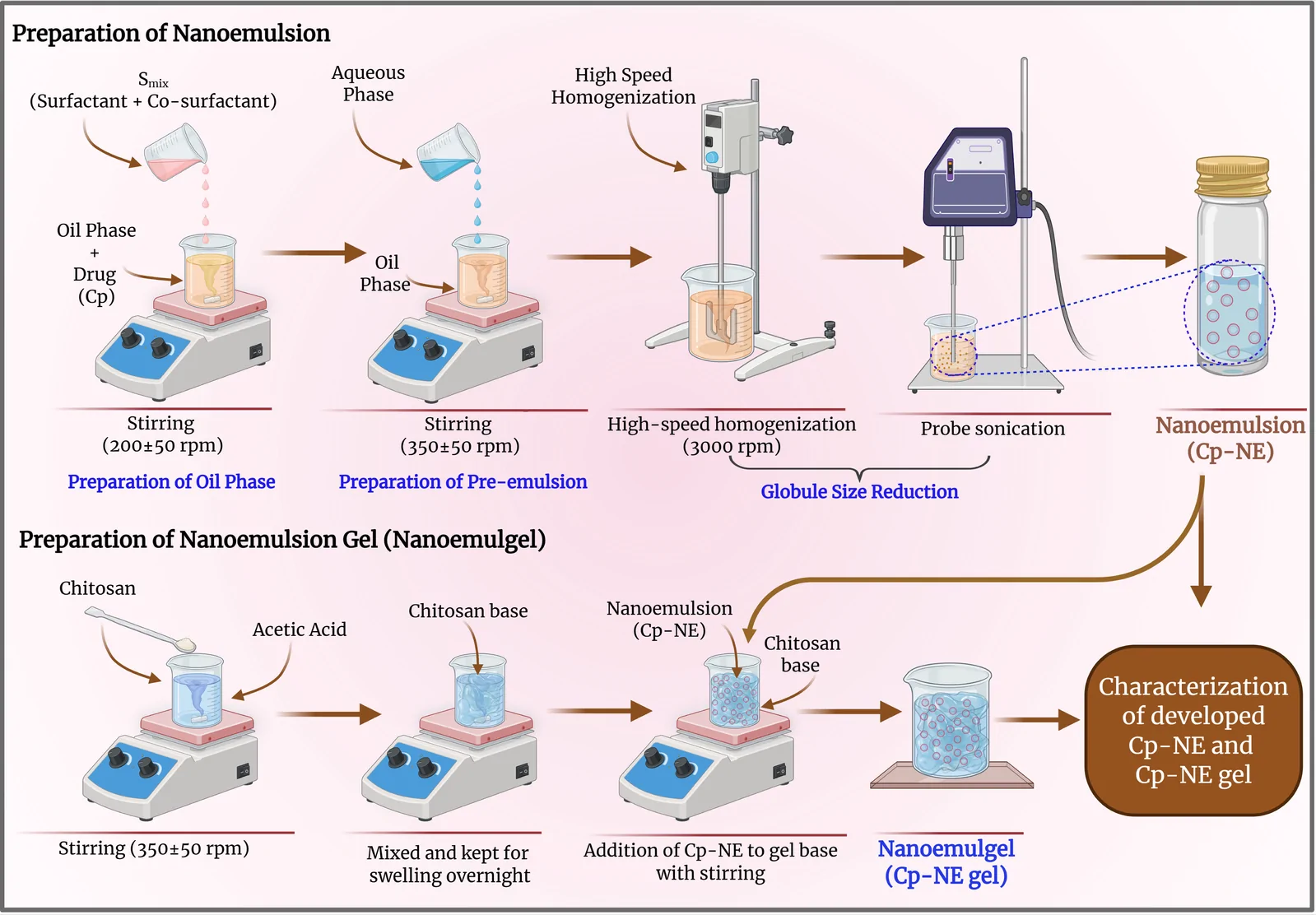
Schematic illustration of the stepwise preparation process for Cp-NE and chitosan-based nanoemulgel *i.e.* Cp-NE gel.

The formulation of Cp-NE was prepared by incorporating the oil phase, consisting of oleic acid, and a *S*_mix_ system containing Tween-80 and PEG-400 in the desired ratio. The optimized formulation consisted of 10% w/w oleic acid, 3.5% w/w *S*_mix_ (Tween-80 : PEG-400, 1 : 1), and distilled water as the continuous phase. The final formulation was developed from the optimum formulation derived through experimentation. An accurate amount of “Cp” was weighed (2 mg) and dissolved in the oil phase by mixing in an agitated manner at ambient temperature (25 ± 2 °C). After that, a continuous aqueous phase was slowly added dropwise to the oil phase during homogenization at 3000 rpm for 01 h in a high-speed homogenizer.^28,29^ These factors together formed a nano-dispersed emulsion system. Later, the NEs formed were further sonicated by using a probe sonicator and stored under controlled conditions (Fig. 1).

#### 2.2.1. Design and optimization using BBD

##### 2.2.1.1. Identifying QTPP, CQAs, and CPP

**Fig. 2:**
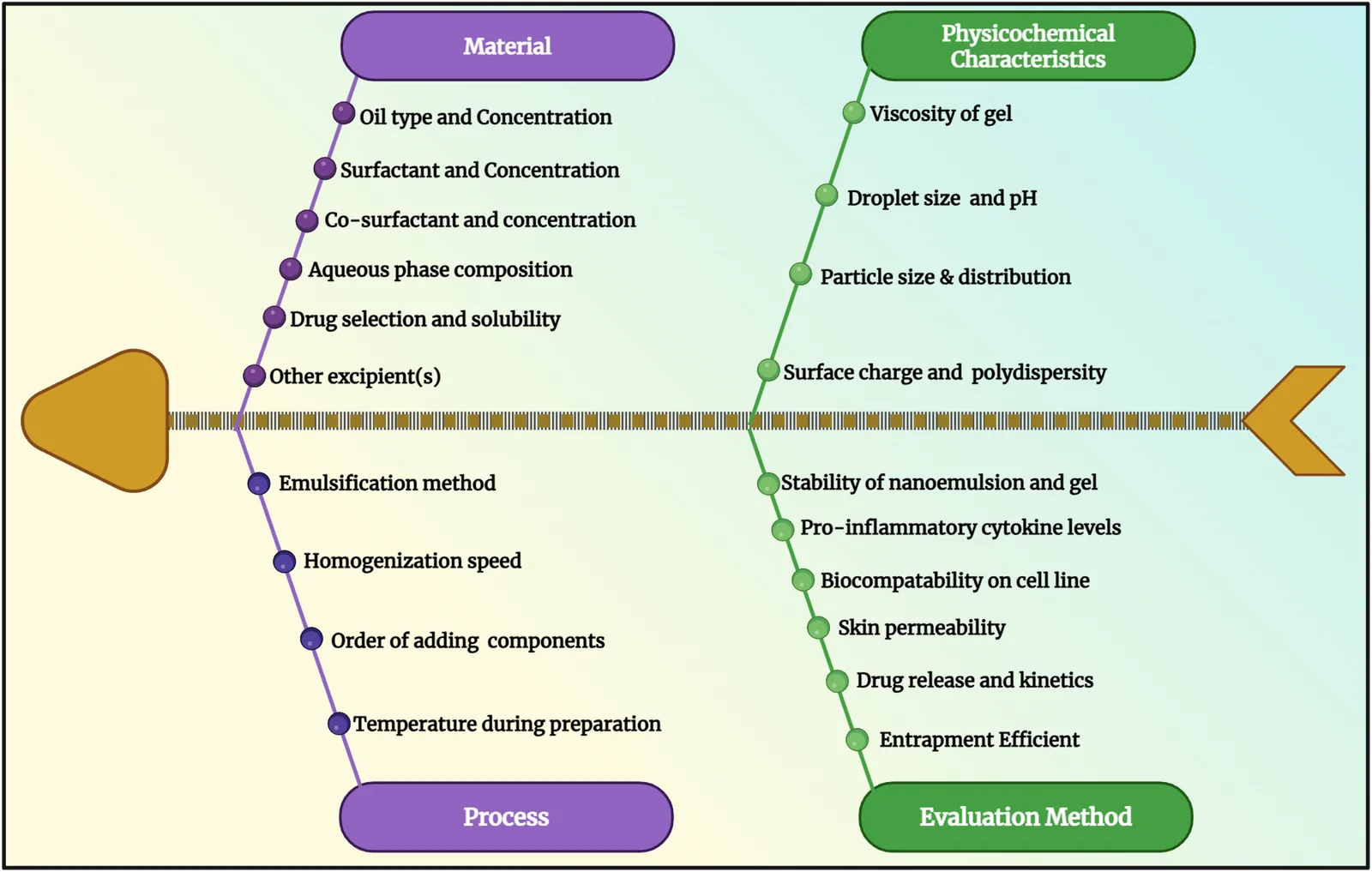
Ishikawa fishbone diagram represents the correlation between the variables in the formulation and the process.

The development of NE formulations began by describing the QTPP using the QBD framework, with the parameters and justifications listed in Table 1. CQAs such as globular size, zeta potential ZP, and EE were identified as key response variables impacting the process. Type of surfactant and its concentration, speed, and quantity of lipids and polymer used are the CPPs. Fig. 2 illustrates the Ishikawa Fishbone diagram outlining the CMA, CPP, physicochemical characteristics, and evaluation parameters affecting the development and optimization of the Cp-NE gel.^30,31^

**Table 1:** Various Independent and dependent variables and constraints

Experimental variables	Levels			Constraints
	Low	Medium	High	
*A* = oil concentration (%)	3	5	10	In range
*B* = surfactant concentration (%)	1.75	2.00	3.5	In range
*C* = homogenization speed (RPM)	1000	2000	3000	In range

**Dependent variables (responses)**				
*X* = EE (%)				Minimum
*Y* = ZP (mV)				Maximum
*Z* = globule size (nm)				Minimum

##### 2.2.1.2. Optimization of Cp-loaded NE using the BBD approach

Response surface methodology (RSM) was implemented using a 3-factor, 3-level BBD to systematically optimize the preparation of Cp-loaded NE for topical application, employing Design-Expert (version 11). This design was selected based on prior evidence and formulation considerations, as it enables robust characterization of quadratic response surfaces *via* R polynomial modeling while maintaining experimental efficiency by reducing the number of runs. Variables-oil concentration (*A*, % w/w), *S*_mix_ concentration (*B*, % w/w), and homogenization speed (*C*, RPM)-affect NEs droplet size, polydispersity, and stability.^32^ The experimental data were evaluated using multiple regression models (polynomial models) to assess the interaction between the CPPs. The statistical analysis used *p*-values to determine the effects of the responses. Process optimization was conducted using a second-order model for the response variables, and the model's adequacy was assessed using experimental and predicted values. 2D and 3D graphs of the interaction were possible with RSM. The oil concentration range (3–10%) was selected based on preliminary pseudo-ternary phase diagram studies that demonstrated stable nanoemulsion formation within this region while maintaining desirable physicochemical characteristics. During optimization, Tween-80 and PEG-400 were maintained at a fixed 1 : 1 ratio (*S*_mix_). Consequently, any change in *S*_mix_ concentration proportionally altered both surfactant and co-surfactant concentrations while preserving their ratio. The aqueous phase was adjusted accordingly to maintain the total formulation composition.

### 2.3. Characterization of Cp-NE

#### 2.3.1. Globule size, PDI, and ZP

The mean globule size and PDI were measured using DLS with a Zetasizer Nano ZS (Malvern Instruments Ltd, UK). For this, samples were diluted 10-fold with distilled water to reduce interactions and scattering, then analyzed at 160° at 25 °C.^33^

#### 2.3.2. Entrapment efficiency

The %EE of the Cp in the NE system was estimated *via* a UV-visible spectroscopic approach at 237 nm. The prepared formulation was spun in a centrifuge machine at 12 000 rpm for 30 min to separate the unentrapped fraction and calculate the EE% by the given formula:^34^
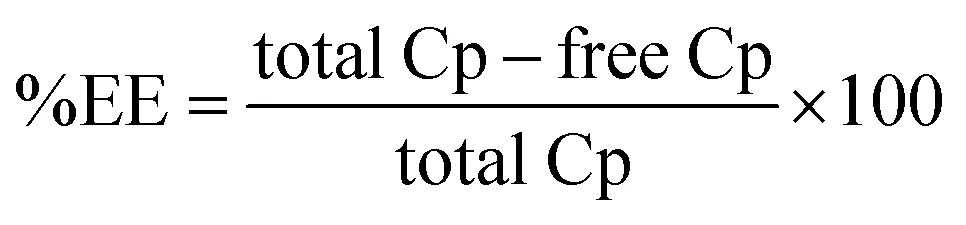


#### 2.3.3. Drug content

The drug content was measured by UV-visible spectrophotometry. 1 mL of Cp-NEs was diluted to 10 mL in ethanol, shaken for 30 minutes at 50 rpm, and then the absorbance was measured at 237 nm against a blank, using a Cp calibration curve.^35^ The drug content was subsequently calculated using the following formula:
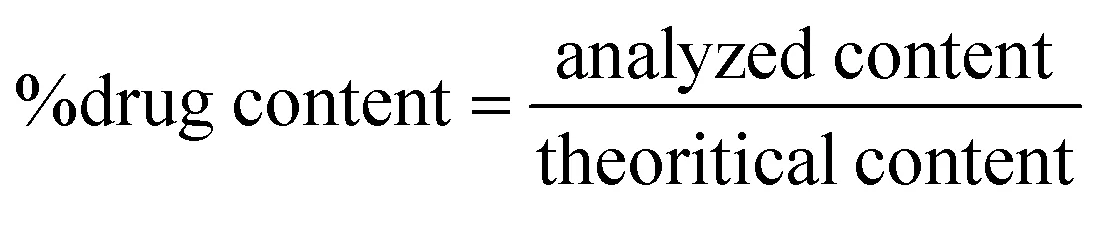


### 2.4. Preparation of nanoemulsion gel

A chitosan gel was formulated by dissolving 2% w/v chitosan in distilled water containing 2% v/v acetic acid. This protonates amino groups, allowing dissolution and forming a clear solution.^36^ The solution was left at RT overnight to allow partial dehydration and solvent evaporation, yielding a semi-solid gel. The Cp-NE formulation was then incorporated into the chitosan gel at a 1 : 1 ratio and stirred for 20 min to prevent phase separation and achieve a uniform distribution of NEs. The formulation pH was then adjusted to 4.5–5.5 with a buffer to match the pH of human skin.^37,38^

#### 2.4.1. Characterization of Cp-NE gel formulation

##### 2.4.1.1. Visual inspection

Visual observation was used to assess the physicochemical properties of the Cp-NE gels, including color, homogeneity, and phase separation. The gels were inspected under sufficient lighting and stored to check for cracking or oil oozing, which could indicate instability.^39^

##### 2.4.1.2. Viscosity

The viscosity of Cp-NE gel was measured with a Brookfield viscometer (Model DV-E) using a 06 No. spindle at 25 °C. About 100 g of gel was placed in a beaker, and the spindle was immersed vertically without touching the bottom or sides.^40,41^

##### 2.4.1.3. Spreadability

Spreadability was measured using a modified sliding board with a wood block and a glass slide. A 20 g load was applied to the upper slide, and the time to complete displacement under this load was recorded. The spreadability index was calculated from this time, the slide displacement length, and the weight^42^ using the following equation:
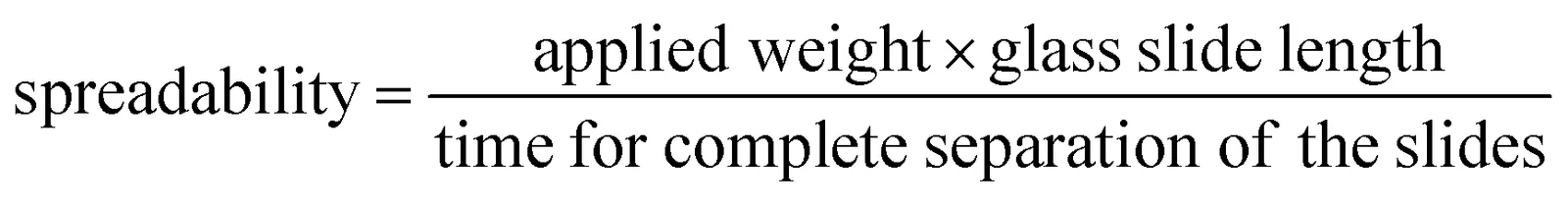


##### 2.4.1.4. Swelling index

1 gm of the gel was placed in an empty basket, and the combined weight of the basket and the gel was determined. A beaker was filled with 10 mL of PBS of pH 5.5, and the basket comprising the gel was partially submerged. The PBS level remained below 10 mL to prevent the gel from dissolving. The basket was immersed to about 1/4th of its depth, and the weight of the basket containing the gel was recorded at various time intervals.^43^ The following equation determined the swelling index:
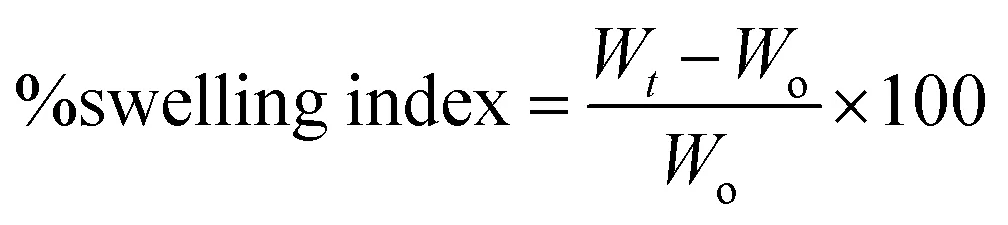
where *W*_*t*_ = swollen gel weight after time *T*; *W*_o_ = initial weight of gel.

##### 2.4.1.5. Drug release and kinetics studies

The release of Cp from the NE and NE-based gel was estimated *via* a dialysis membrane method. The receptor medium (200 mL) was a hydroalcoholic PBS (10% ethanol, pH 5.5) solution that ensured suitable solubility and sink conditions at 37 ± 0.5 °C, and stirring was maintained at 100 rpm to mimic physiological conditions. An appropriate amount of the sample was cautiously pipetted onto the dialysis membrane bag, then the bag was tightly closed and submerged in the release medium. Samples were withdrawn at fixed time points (1, 2, 4, 6, 8, and 12 hours) and changed with an identical volume of release medium to conserve a constant volume and sink condition. The drug release was measured at 237 nm.^44,45^ The release data for the samples were further analyzed using release kinetic models to determine the release mechanism. The fit of the model was assessed using the coefficient (*R*^2^), and the higher the value, the better the fit.^46,47^

##### 2.4.1.6. *In vitro* permeation studies

The *in vitro* tests evaluating skin permeation properties were performed using Franz diffusion cells, in which skin samples collected from a local slaughterhouse (Aslam Meat and Chicken Supplier – Vapariyo Ka Mohla, Rajgarh – Alwar Rd, Katariyo Ka Moholla, Pilani, Rajasthan) were placed between the donor and receiver chambers. The medium used comprised PBS solution supplemented with 10% ethanol, adjusted to pH 5.5, and kept agitated at 37 ± 0.5 °C and 200 rpm. The formulation under examination was loaded into the donor chamber, and samples of 0.5 mL were drawn from the receiver chamber at specified time points. After each sampling procedure, the same amount of medium was replenished into the recipient chamber to preserve sink conditions. The tests were repeated in triplicate to ensure the reproducibility of the results. Chromatographic analysis of samples was performed to obtain the drug's skin permeation kinetic profile.^48^

##### 2.4.1.7. Biocompatibility assessment

The cytotoxicity of the Cp, Cp-NE, and Cp-NE gel was assessed using HaCaT cells obtained from the laboratory of Professor Gautam Singhvi, BITS Pilani, Pilani Campus, Rajasthan, India. Cell cultures were propagated in DMEM supplemented with 10% and 1% Fetal Bovine Serum, respectively, and penicillin–streptomycin to promote optimal cell growth and survival. These cells were seeded at a concentration of 1 × 10^5^ cells per mL and cultured at 37 °C and 5% CO_2_ for 24 hours to achieve adhesion and stabilization. Following the incubation period, the free drug, Cp-NE, and the Cp-NE gel were added at various concentrations and further incubated for an additional hour. Viability was evaluated by adding 0.5 mg mL^−1^ MTT solution to each well and incubating for 4 h to reduce MTT to formazan crystals. The insoluble formazan crystals were separated and solubilized in DMSO, and the resulting solution was quantitatively evaluated spectrophotometrically at 570 nm to evaluate the % cell viability relative to the untreated control.^49,50^

##### 2.4.1.8. *In vitro* cytokine assay

RAW264.7 murine macrophages obtained from the laboratory of Professor Gautam Singhvi, BITS Pilani, Pilani Campus, Rajasthan, India, were seeded into 6-well plates at a density of 3 × 10^5^ cells per well and incubated for 24 h at 37 °C in an atmosphere containing 5% CO_2_ under standard culture conditions. After incubation, the cells were treated with different formulations, including free drug Cp, Cp-NE, and Cp-NE gel, at concentrations of 10, 20, 40, and 80 µg mL^−1^ in the presence of lipopolysaccharide (LPS, 5 µg mL^−1^) for an additional 24 h. Untreated cells served as the normal control, whereas cells treated with LPS alone were considered the inflammatory control. Following treatment, the culture supernatants were collected and centrifuged to remove cellular debris. Pro-inflammatory cytokines, including IL-1β and TNF-α, were measured using standard commercially available ELISA kits according to the manufacturer's instructions. In brief, standards and samples were added to antibody-specific pre-coated ELISA plates and incubated at 37 °C. The unbound substances were washed, enzyme-linked detection antibodies were added, and then the substrate solution was added to develop color. The reaction was stopped with the stop solution, and absorbance was measured at 450 nm using a microplate reader, and cytokine concentrations were determined.^51,52^

##### 2.4.1.9. Stability studies

The stability of the formulations was evaluated by storing samples at 2 controlled temperatures: 5 ± 3 °C and 25 ± 2 °C/60 ± 5% RH. Samples were collected at predetermined intervals and analyzed for drug content and globule size. The chosen storage temperatures, 5 ± 3 °C and 25 ± 2 °C/60 ± 5% RH, reflect the storage conditions that are usually considered while performing stability tests on topical nanoformulations.^53^ The accelerated temperatures >30 °C have not been used due to the susceptibility of NEs to thermodynamic instabilities such as particle coalescence, phase separation, and drug degradation. Thus, these temperatures allowed carrying out a proper study of physical stability and drug release from the formulation.

### 2.5. Statistical analysis

The analysis was performed in triplicate, and the data are presented as means ± SD. The analysis of differences between groups was done statistically through the ANOVA test. For the design of experiments, Design-Expert software (version 11) was used, and OriginPro was used for statistical analysis and graphical presentation of results. Results were regarded as statistically significant at *p*-value <0.05.

## 3. Results and discussion

### 3.1. Pre-formulation screening of NE components

**Fig. 3:**
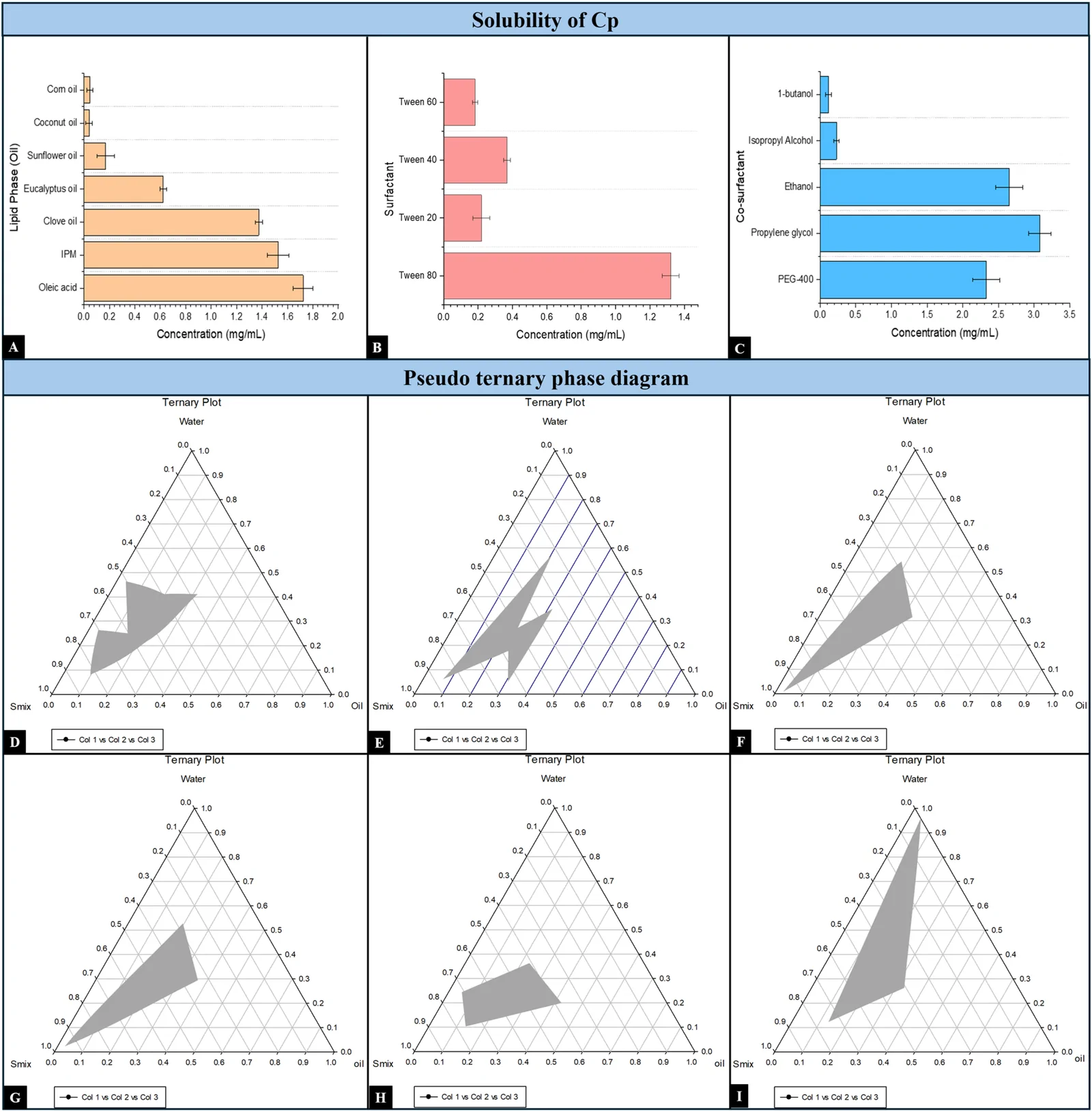
Solubility of Cp in [A] oil, [B] surfactant, and [C] co-surfactant; (ii) phase diagram (D-I).

The formulation included oleic acid as the oil phase, *S*_mix_ (Tween-80 and PEG-400, 1 : 1), and chitosan as the gelling agent. Initially, the drug's solubility in various oils, surfactants, and co-surfactants was determined. The solubility of Cp in the oils was highest in oleic acid (1.723 ± 0.67 mg mL^−1^) and IPM (1.527 ± 0.74 mg mL^−1^), indicating a comparatively higher solubilizing capacity. In contrast, Tween 80 (1.32 ± 0.53 mg mL^−1^) demonstrated the most effective surfactant. Propylene glycol (3.08 mg mL^−1^), followed by ethanol (2.65 mg mL^−1^) and PEG-400 (2.33 mg mL^−1^), indicating their suitability as co-surfactants [Fig. 3(A–C)].

### 3.2. Pseudo-ternary phase diagrams

To assess the optimal ratio of oil, *S*_mix_, and water to obtain a stable NE system, phase diagrams were generated. The pseudo-ternary phase diagram was plotted to determine the most optimal compositions of oil, *S*_mix_, and water for the formation of stable NE. The formation of transparent, isotropic phases marked the nanoemulsion region, where the area size depended on the emulsification ability of the chosen *S*_mix_ ratio. Hence, the phase diagram became an efficient tool for determining the best composition for further formulation development.^54,55^ The effect of various ratios of *S*_mix_, including Tween 80 : PG and Tween 80 : PEG 400 in 1 : 1, 2 : 1, and 3 : 1 ratios, along with IPM, oleic acid, and castor oil as oils, was investigated to delineate the NE region (Fig. 3. Based on observations, formulations demonstrating turbidity were considered potential for forming NE. As illustrated in Fig. 3, the area of NEs progressively increased from Fig. 3D–G, reflecting the fact that better emulsification was achieved at higher ratios of *S*_max_ because of improved interfacial stabilization and low interfacial tension. Nonetheless, an additional increment in the Smix ratio (Fig. 3H and I) led to a decreased area of NE formation, indicating that the high surfactant concentration did not improve NE formation. According to the findings, higher *S*_mix_ ratios and appropriate oil amounts contributed to NE generation by lowering interfacial tension and increasing stability. In comparison, the combination of Tween 80 with PEG 400 and oleic acid/PG yielded a more stable NE system than castor oil.^56^

### 3.3. Optimization by BBD

**Fig. 4:**
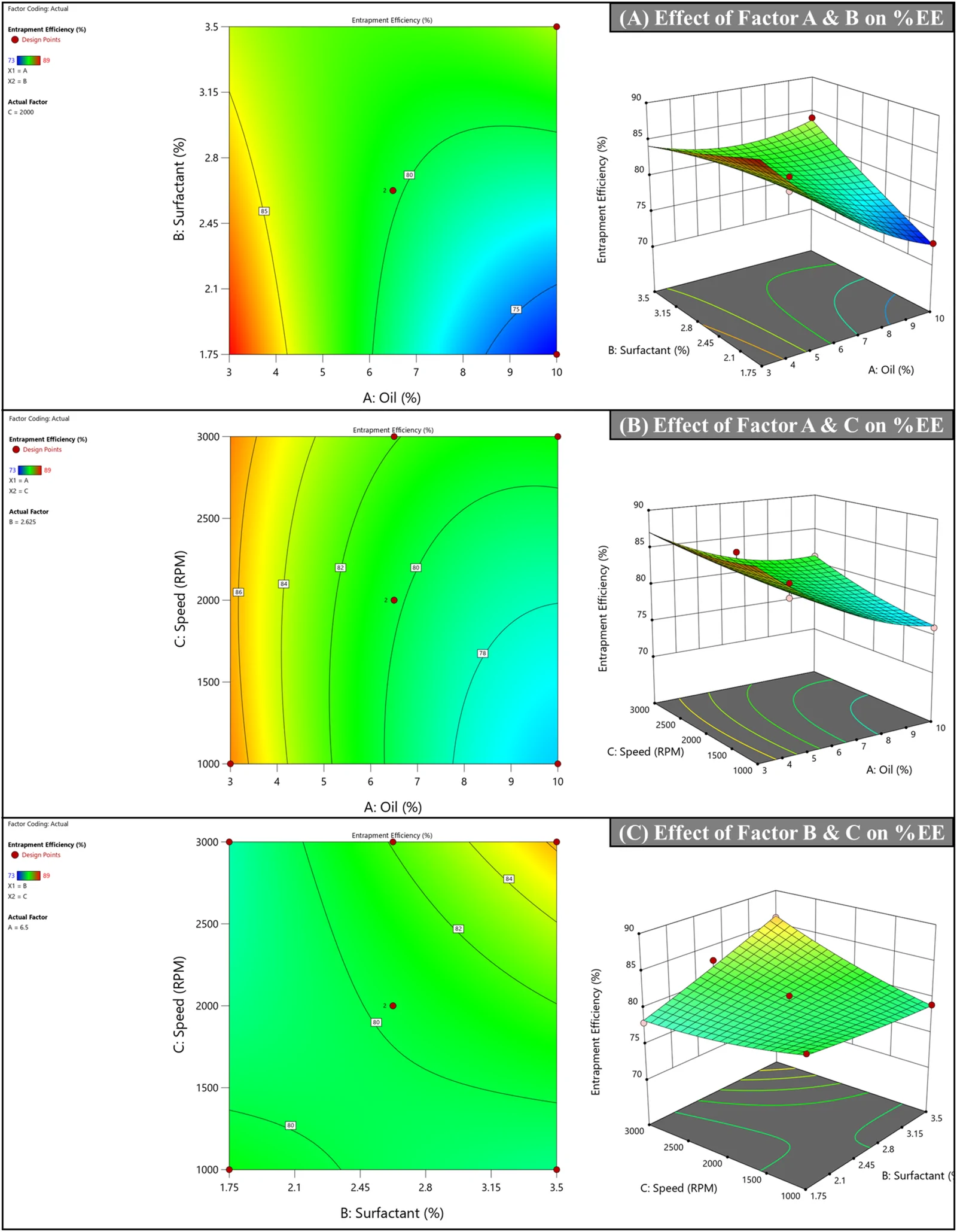
Response surface plots illustrating the effects of factors A, B, and C on the %EE of Cp-NE: (A) factors A and B; (B) factors A and C; and (C) factors B and C.

**Fig. 5:**
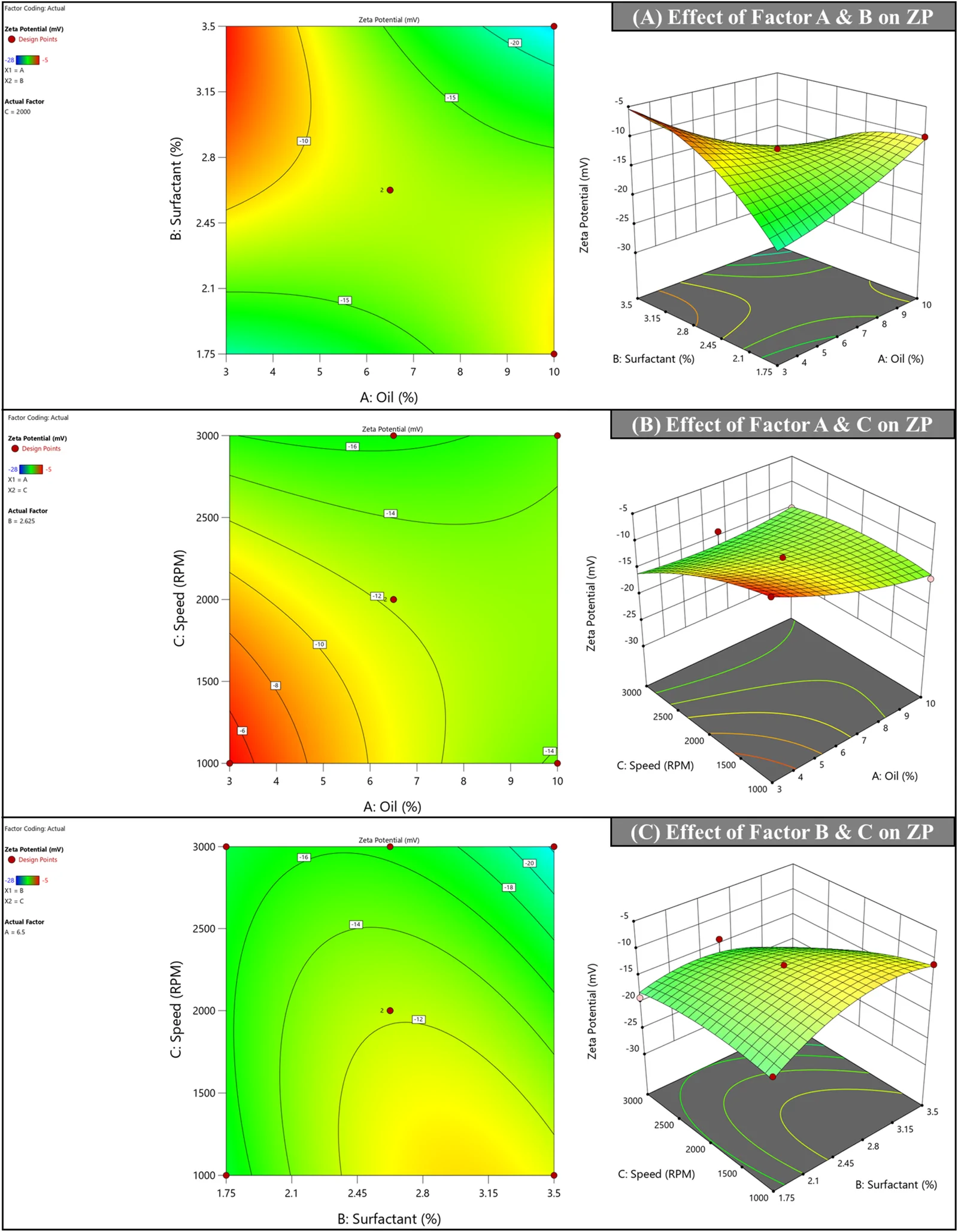
Response surface plots illustrating the effects of factors A, B, and C on the ZP of Cp-NE: (A) factors A and B; (B) factors A and C; and (C) factors B and C.

**Fig. 6:**
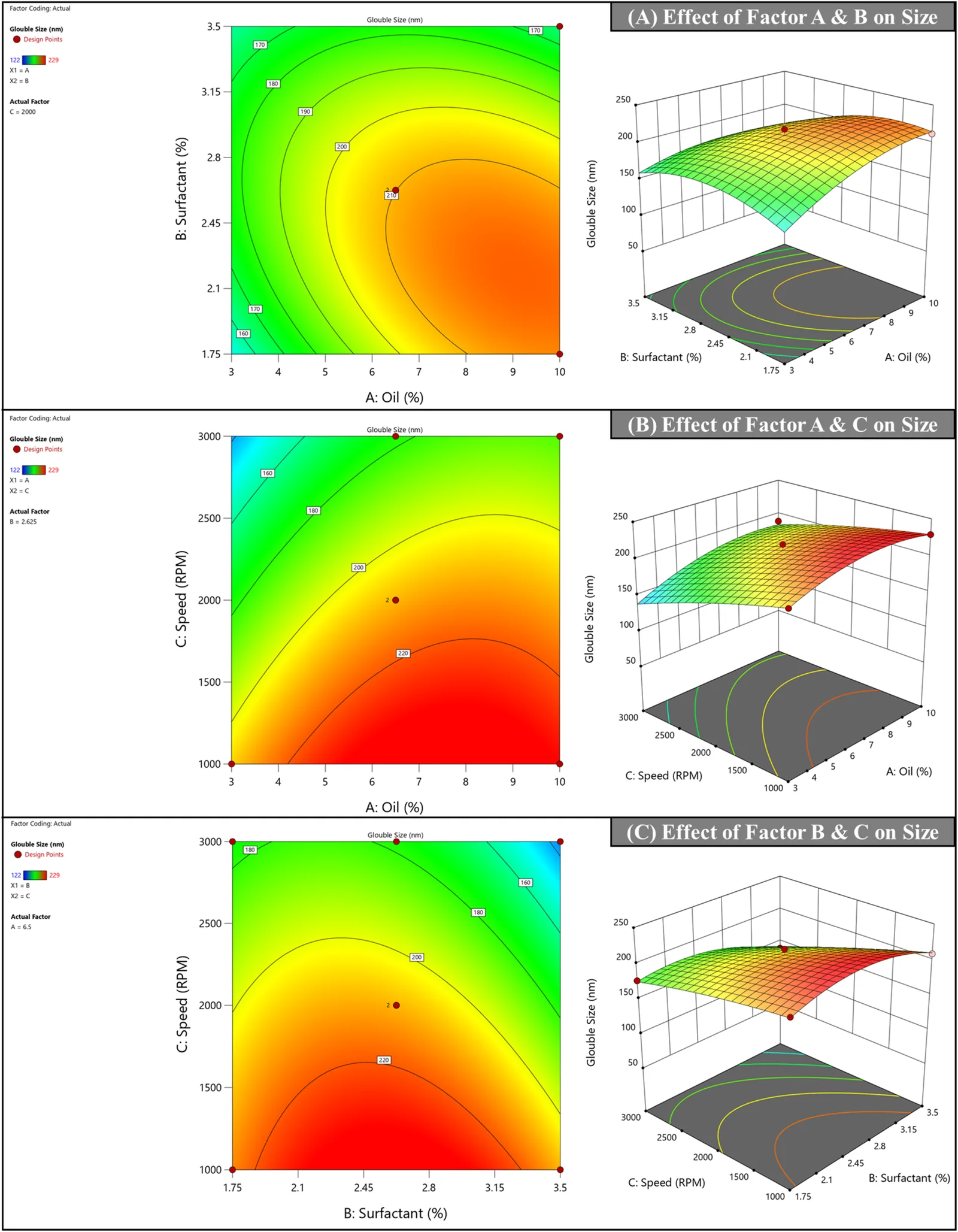
Response surface plots illustrating the effects of factors A, B, and C on the globule size of Cp-NE: (A) factors A and B; (B) factors A and C; and (C) factors B and C.

The phase diagrams guided the selection of formulation variables for new emulsions. A BBD assessed oil (*A*), surfactant (*B*), and homogenization speed (*C*) effects on %EE (*X*), ZP (*Y*), and globule size (*Z*). Significant effects from surfactant and speed improved emulsification. The optimized formulation with 10% oil, 3.5% surfactant, and 3000 rpm achieved 89% EE, a ZP of −28 mV, and a globule size of 122 nm, demonstrating the BBD model's effectiveness (Table 2). The %EE of Cp-NE formulations was found to be between 73 and 89%, and the maximum %EE was observed in the run 10 formulation, having 10% oil, 3.5% surfactant, and 3000 rpm homogenization speed. The drug entrapment was significantly increased by higher surfactant concentration and homogenization speed, which were probably due to reduced interfacial tension and improved droplet stabilization. The interaction effect of surfactant concentration and homogenization speed on %EE was also confirmed by the response surface plot (Fig. 4). As depicted in Fig. 4A, a higher oil concentration caused a decrease in %EE, whereas a higher surfactant concentration caused slight improvements in %EE, indicating the presence of an optimum surfactant concentration that was able to stabilize the droplets. In Fig. 4B, an increase in homogenization speed led to higher %EE, especially at low oil concentrations, which could be associated with the formation of smaller droplets and better drug encapsulation within them. Likewise, an increase in the surfactant concentration and homogenization speed in Fig. 4C caused gradual improvements in %EE by stabilizing the NE formulation. The zeta potential values varied between −5 mV and −28 mV, suggesting the formation of physically stable nanoemulsion droplets bearing a negative charge. The optimized formulation showed the highest magnitude of zeta potential (−28 mV), indicating better electrostatic stabilization and a lesser tendency for aggregation. As shown in Fig. 5, the higher concentration of surfactant and homogenization speed significantly contributed to the improved formulation stability. In Fig. 5A, an increase in the concentration of both oil and surfactant led to the negative zeta potentials of the NE formulation, which is indicative of increased electrostatic stabilization of the nanoemulsion droplets. As shown in Fig. 5B, an increase in homogenization speed also led to more negative zeta potentials, especially at moderate oil concentrations, due to the formation of smaller droplets with improved surface charge distribution. In addition, as shown in Fig. 5C, the increase in both surfactant concentration and the homogenization speed also contributed to comparatively high negative values of the zeta potential. The size of the prepared formulations ranged from 122 nm to 229 nm. The increase in surfactant concentration and homogenization speed was very effective in reducing globule size by disrupting and stabilizing droplets during emulsification. NE with a smaller globule size shows better stability and permeation. The surface response plot in Fig. 6 confirmed the significant effect of formulation variables on globule size reduction. It is evident from Fig. 6A that globule size increased with increasing oil concentration, but higher surfactant concentration decreased globule size due to better interfacial coverage and stabilization of newly formed droplets. Fig. 6B showed that with an increase in homogenization speed, globule size decreased, especially at low to moderate oil concentration, due to the greater shearing forces available for effective droplet breaking. In addition, Fig. 6C showed that the effect of increased surfactant concentration and homogenization speed led to small globule sizes due to the better emulsification process and droplet stabilization.

**Table 2:** Experimental runs for Box–Behnken design for optimization of Cp-NE

Run	Factor			Response		
	*A*: oil (%)	*B*: surfactant (%)	*C*: speed (RPM)	%EE	ZP (mV)	Globule size (nm)
1	6.5	1.75	3000	78	−19	177
2	10	2.625	3000	81	−15	189
3	6.5	2.625	2000	79	−14	202
4	10	2.625	1000	76	−15	229
5	6.5	2.625	2000	81	−12	218
6	6.5	3.5	3000	86	−23	140
7	10	1.75	2000	73	−10	212
8	6.5	2.625	3000	83	−14	172
9	6.5	1.75	1000	81	−17	216
10	10	3.5	3000	89	−28	122
11	6.5	3.5	1000	79	−11	209
12	10	3.5	2000	84	−22	165
13	3	2.625	1000	87	−5	207

The ANOVA analysis from the BBD results disclosed that all three responses, *i.e.*, %EE, ZP, and globule size, were significant models as indicated by the *p*-values and *F*-values (Table 3). For the %EE, the model was significant (*p*-value = 0.0125, *F*-value = 23.42). Among the factors, *A*, *AB*, and *BC* interactions showed a notable effect (*p* < 0.05). The lack-of-fit for %EE was non-significant (*p*-value = 0.7662), confirming the model's suitability. Eqn (1). For the ZP, the *p*-value was 0.0389, and the *F*-value was 10.57, confirming the model's significance. In this, the interaction terms *C* and *AB* showed significant effects (*p*-values of 0.0614 and 0.0293, respectively). Whereas the other interaction effects were not statistically significant (*p*-values >0.05). The lack-of-fit was non-significant (*p*-value = 0.3828), indicating a good fit of the model. For globule size, the model showed strong significance (*p*-value = 0.0207, *F*-value = 16.51). The linear term *C* and quadratic term *B*^2^ were observed to be most significant with a *p*-value of 0.0066 and 0.029, respectively. The lack of fit persisted non-significantly (*p* = 0.7195), further validating the model. The analysis indicated that specific factors and their interactions had a key role in optimizing formulation parameters; in contrast, the developed models were found to be reliable and suitable for prediction within the studied design space. The correlation between the independent and dependent variables was modeled using polynomial equations. These include linear, interaction, and quadratic terms to acquire both individual and combined effects of the variables. The equation expressed in terms of coded variables is useful for predicting the response to different values of the variables. High values of the variables are represented by +1, while low values are represented by −1. The coded equation is important for determining the magnitude of each variable's influence through its coefficient. The regression polynomial equation for the BBD method:

1 %EE = +80.26 − 24.17*A* + 1.50*B* + 1.22*C* + 3.92*AB* + 1.20*AC* + 2.46*BC* + 1.96*A*^2^ + 0.1931*B*^2^ + 0.6345*C*

2 ZP = −12.23 − 2.03*A* + 0.5000*B* − 2.88*C* − 6.80*AB* + 2.58*AC* − 2.65*BC* + 1.13*A*^2^ − 3.64*B*^2^ − 1.39*C*^2^

3 Glouble size = +209.91 + 16.76*A* − 11.00*B* − 28.38*C* − 15.08*AB* + 5.81*AC* − 8.79*BC* − 16.33*A*^2^ − 21.74*B*^2^ − 4.00*C*^2^

**Table 3:** ANOVA summarizing the impact of independent variables *i.e., A*: oil; *B*: surfactant; *C*: speed, on responses *i.e.,* %EE, ZP, and globule size

Response 1: entrapment efficiency												
Source	Model	*A*	*B*	*C*	*AB*	*AC*	*BC*	*A* ^2^	*B* ^2^	*C* ^2^	Residual	Lack of fit
Sum of squares	239.36	25.24	9	7	23.36	2.3	26.66	4.09	0.1013	1	3.41	1.41
d*f*	9	1	1	1	1	1	1	1	1	1	3	2
Mean square	26.6	25.24	9	7	23.36	2.3	26.66	4.09	0.1013	1	1.14	0.7034
*F*-Value	23.42	22.23	7.93	6.16	20.57	2.02	23.48	3.61	0.0892	0.8805		0.3517
*p*-Value	0.0125	0.0181	0.067	0.0891	0.0201	0.2501	0.0168	0.1538	0.7847	0.4173		0.7662

**Response 2: zeta potential**												
Sum of squares	432.66	6	1	38.84	70.32	10.67	30.93	1.37	36.02	4.82	13.65	11.65
d*f*	9	1	1	1	1	1	1	1	1	1	3	2
Mean square	48.07	6	1	38.84	70.32	10.67	30.93	1.37	36.02	4.82	4.55	5.82
*F*-Value	10.57	1.32	0.2198	8.54	15.46	2.35	6.8	0.3012	7.92	1.06		2.91
*p*-Value	0.0389	0.334	0.6712	0.0614	0.0293	0.2231	0.0799	0.6213	0.0671	0.379		0.3828

**Response 3: globule size**												
Sum of squares	12 243.66	407.23	484	3782.84	345.99	54.07	340.62	285.73	1283.91	39.78	247.27	119.27
d*f*	9	1	1	1	1	1	1	1	1	1	3	2
Mean square	1360.41	407.23	484	3782.84	345.99	54.07	340.62	285.73	1283.91	39.78	82.42	59.63
*F*-Value	16.51	4.94	5.87	45.9	4.2	0.656	4.13	3.47	15.58	0.4826		0.4659
*p*-Value	0.0207	0.1127	0.0939	0.0066	0.1329	0.4773	0.135	0.1595	0.029	0.5372		0.7195

#### 3.3.1. Model adequacy (residuals *vs.* run) and perturbation analysis

**Fig. 7:**
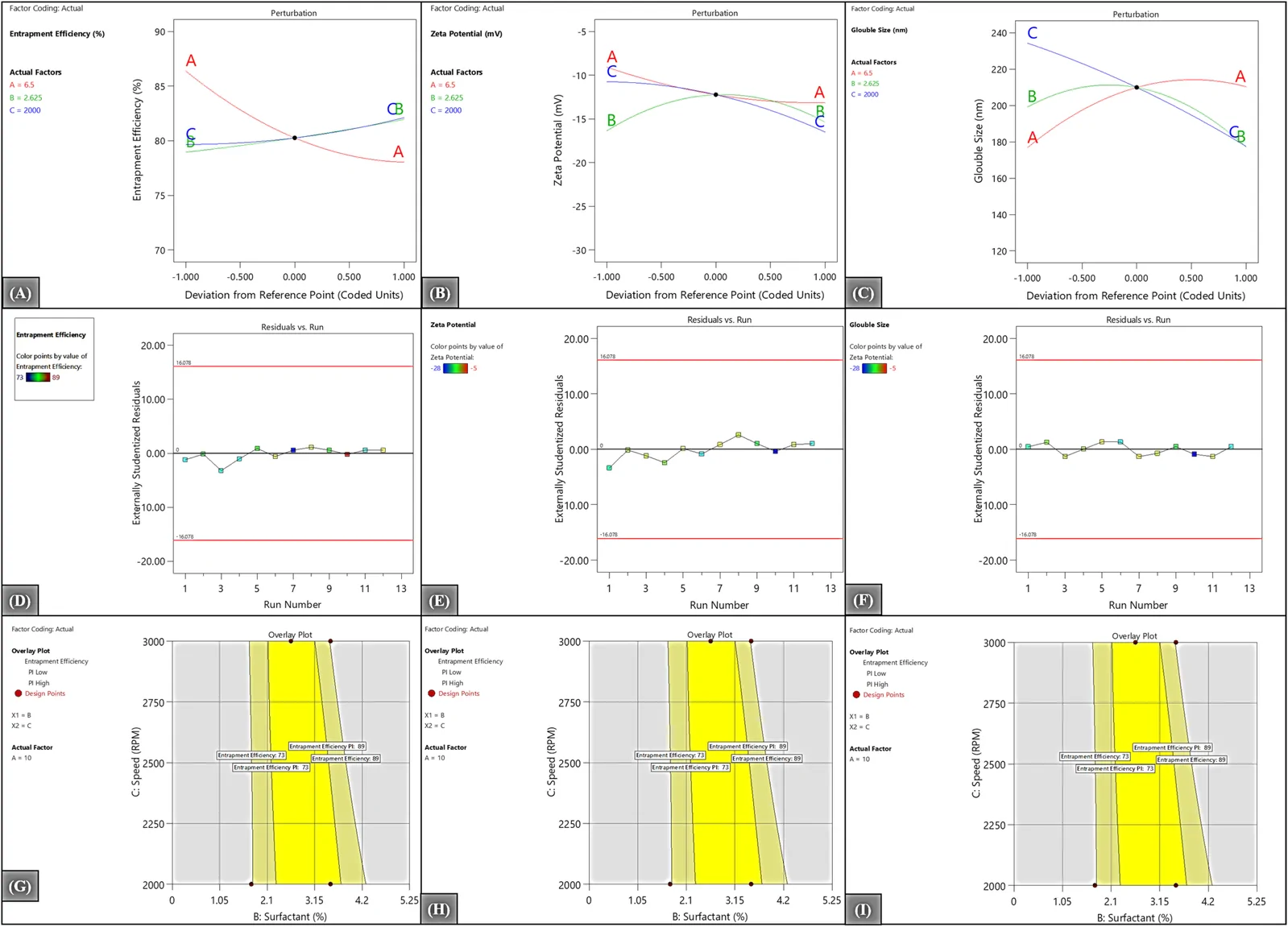
Studentized residuals *vs.* run plots (A–C); perturbation analysis (D–F) and overlay plot indicating the design space (G–I).

Across %EE, ZP, and globule size, the externally studentized residuals *vs.* run plot was randomly scattered around zero and lay within the control limits, indicating no outliers and confirming independence of errors.^57^ All residuals observed within the acceptable control limits, *i.e.*, ±16.08, with no outliers or influential points observed. This represented good model fit, constant variance, and no systematic bias. Therefore, the developed models were statistically acceptable and reliable for prediction within the studied design space (Fig. 7A–C). Perturbation analysis indicated that %EE was mainly governed by factor *A*, showed a strong inverse relationship, while factors *B* and *C* exerted mild positive effects with relatively lower influence (Fig. 7D). ZP is affected in a nonlinear manner by all variables, with factor *C* having the strongest impact by shifting values towards more negative levels, factor *B* showed an initial increase before stabilizing, and factor *A* represented a slight decreasing trend (Fig. 7E). Globule size was also notably dependent on factors, particularly *C*, which reduced particle size at higher levels, while *A* raised size and *B* showed a quadratic response with an optimum near the center (Fig. 7F).

### 3.4. Physicochemical characterization of NEs

**Fig. 8:**
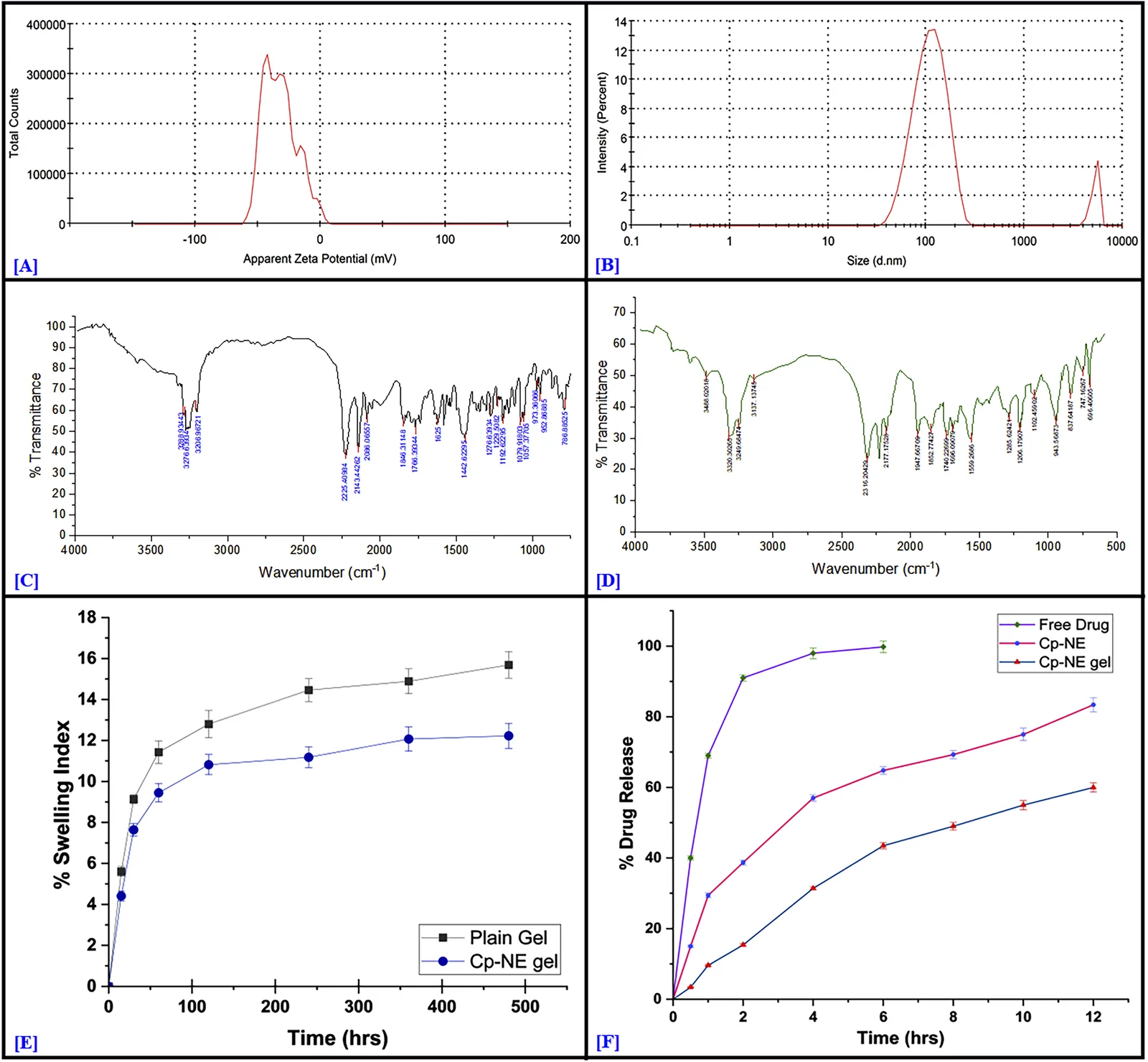
[A] ZP; [B] PDI; FTIR of [C] Cp-NE; [D] Cp-NE gel; [E] swelling index; [F] *in vitro* drug release.

The Cp-NE showed a small globule size of 122.5 nm. Additionally, the observed ZP of −28 mV (Fig. 8A and B) indicated good colloidal stability, which is better for reducing the likelihood of particle aggregation over time. The PDI value of 0.508 ± 0.08 indicated a moderate width of the size distribution, thereby validating the product's uniformity. Cp-NE gels had a pH of 6.13 ± 1.03. Viscosity is an imperative for determining the rate of drug release because a high viscosity decreases the rate of drug release. The viscosity of the NE gel was 6799 ± 15.52 cps, while that of the plain gel was 3835 ± 25.57 cps, indicating a higher viscosity due to the contributions of chitosan and oleic acid. The FTIR spectrum of Cp confirms the presence of key functional groups. The absorption at 1766 cm^−1^ indicates C=O stretching of the ketone, while peaks at 3276 and 3206 cm-1 show O–H stretching of the alcohol group. A band at 1666 cm^−1^ suggests a C–F bond, and absorption at 1846 cm^−1^ indicates C–Cl stretching. Peaks at 1079 cm^−1^ and 1010 cm^−1^ correspond to ether and alcohol bending. Additionally, 1442 cm^−1^ confirms C=C stretching [Fig. 8C]. The FTIR spectrum of the Cp-NE gel shows key peaks that confirm both the drug and the polymer components. A broad band at 3488.02 cm^−1^ indicates O–H stretching, suggesting hydrogen bonding from chitosan and clobetasol. Peaks at 3320.3 and 3137.13 cm^−1^ are due to hydroxyl or amine groups. The peaks at 1740.22 and 1696.09 cm^−1^ confirm C=O stretching of ketone and ester groups. Bands at 1285.62 correspond to C–O–C, indicating ether [Fig. 8D].

### 3.5. Spreadability

Spreadability is one of the key physicochemical parameters that determines the ease of application, distribution of the drug and patient acceptability of the topical gel formulations. An optimal formulation must have a high level of spreadability to facilitate easy application to the affected areas, yet retain sufficient consistency to remain in place during application. To evaluate the spreadability of the preparations, Blank NE gel had a spreadability of 22.7 ± 1.05 g cm s^−1^, while optimized Cp-NE gel demonstrated significantly higher value *i.e.* 25.3 ± 1.81 g cm s^−1^. Improvement in spreadability of Cp-NE gel was determined by the fact that optimized NE-gel matrix provided better balance between viscosity and flowability of the product. Improved spreading property assisting applying the formulation easily and uniformly without causing additional shearing forces. Therefore, there will be good contact between the drug and the skin, which results in an increase in the contact area. It is expected that such properties will improve drug deposition, prolong its residence time, and enhance the efficacy of clobetasol propionate treatment for psoriasis. Swelling index.

Over 480 min, the swelling index of the plain gel increased 15.7-fold, while the NE gel increased 12.2-fold. The NEs gel reduced swelling may be due to emulsified oil droplets and surfactants that restrict water uptake and matrix expansion. This comparison assesses hydration properties, key parameters affecting drug release, and skin residence time [Fig. 8E]. The swelling index of the NE gel is important for regulating drug release by controlling water uptake, hydration of the gel matrix, and diffusion of the NE globules within the gel. The higher swelling observed in the NE-gel increased the hydration of the polymeric matrix, enabling effective diffusion of the NE containing the drug. Thus, the swelling property of the NE-gel led to efficient drug release.^58^

### 3.6. Release analysis and kinetics

**Fig. 9:**
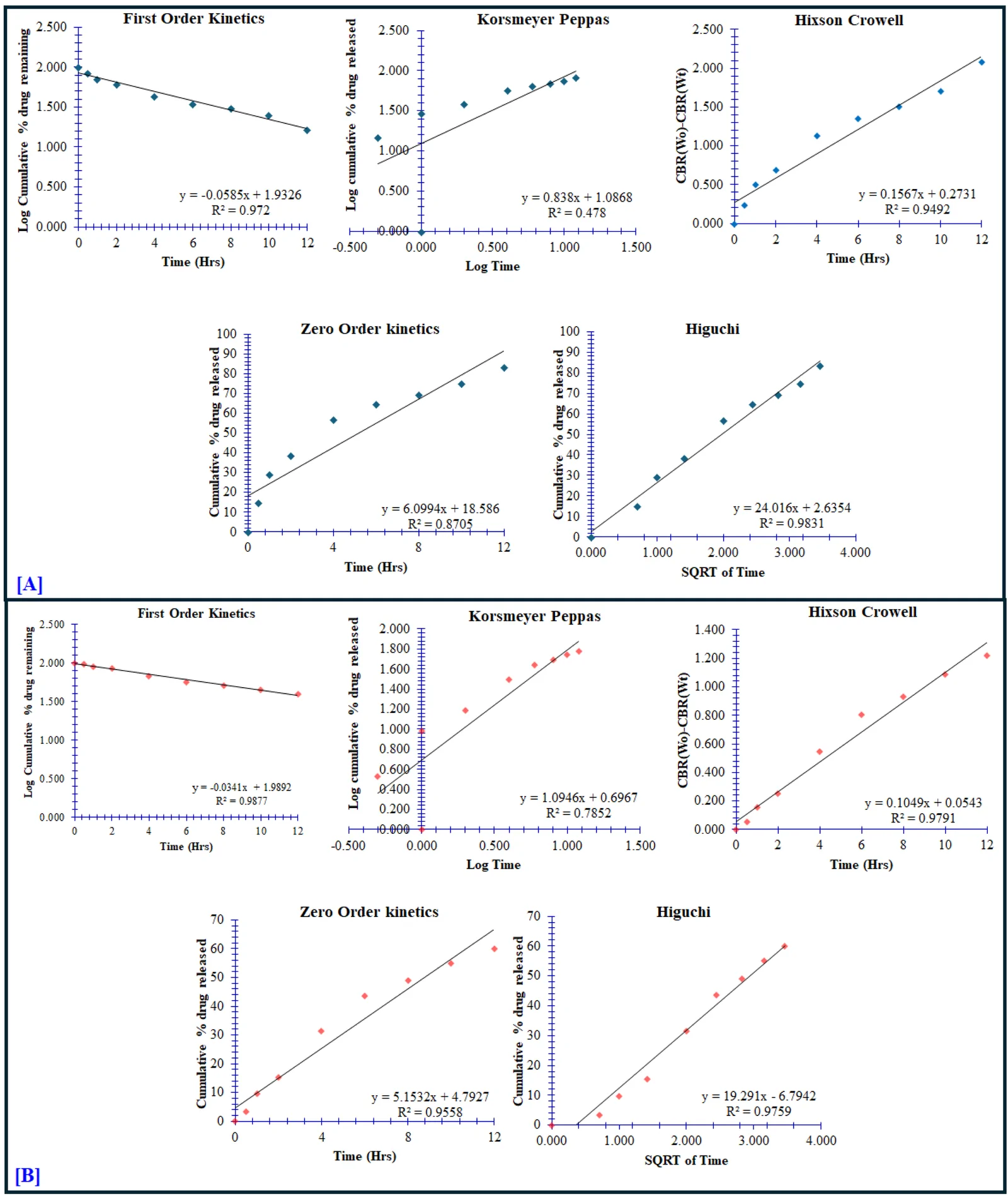
Drug release kinetics of [A] Cp-NE; [B] Cp-NE gel.

*In vitro* release profiles showed considerable variation amongst the different formulations. Free drug showed instant release, releasing 91% in 02 h and reaching maximum release at 06 h. However, in the case of the Cp-NE formulation, 83.4% cumulative release was observed at 12 h, whereas the Cp-NE gel showed prolonged release. At 0.5 h, the free drug and Cp-NE released approximately 11.8 and 4.4-fold more than the Cp-NE gel. After 12 h, both the Cp-NE and free drug showed 1.39- and 1.67-fold higher release than the Cp-NE gel, indicating that NE integration in the gel significantly modulated drug release, supporting its potential for transdermal delivery [Fig. 8F]. Kinetic evaluations for both Cp-NE and Cp-NE gel included zero-order, first-order, Higuchi, Hixson–Crowell, and Korsmeyer–Peppas equations. The Higuchi model provided the best fit for the Cp-NE with an *R*^2^ of 0.9831, indicating diffusion control. The Cp-NE gel showed the best fit, with an *R*^2^ of 0.9492, but also lower regression values, suggesting additional diffusion retardation in the matrix [Fig. 9A]. Also, the Cp-NE gel displayed a good fit to the first-order kinetic model (0.9064), indicating that diffusion is partially controlled in a viscous chitosan matrix [Fig. 9B]. For the Cp-NE and Cp-NE gel samples, the correlation coefficient was low for zero-order kinetics (0.8705 and 0.8371, respectively). The correlation for both samples using the Korsmeyer–Peppas kinetic model was also very poor (0.478 for Cp-NE and 0.693 for Cp-NE gel).

### 3.7. *In vitro* skin permeability study

**Fig. 10:**
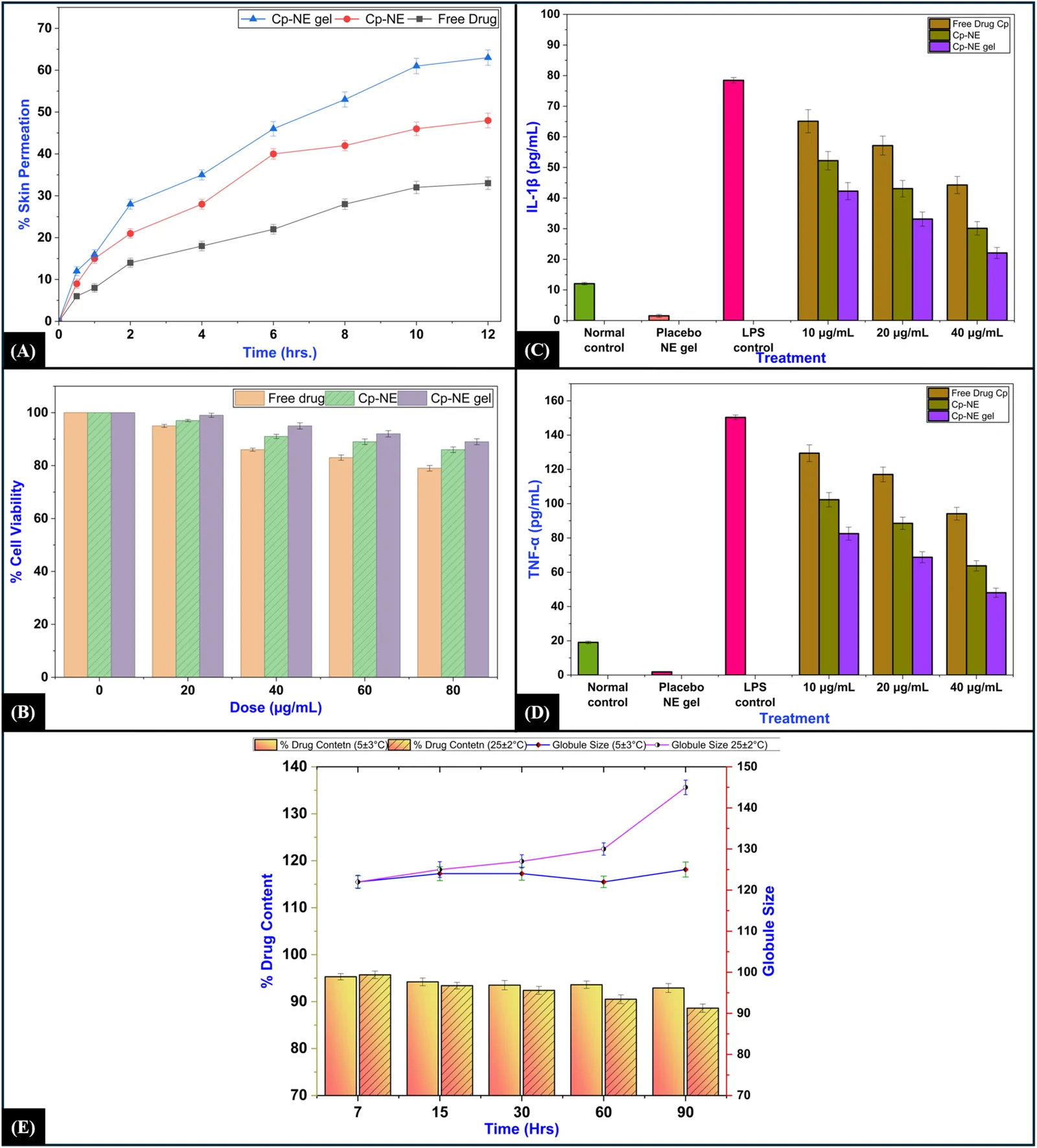
[A] *In vitro* skin permeability; [B] cell viability study; cytokine inhibition by free Cp, Cp-NE, and Cp-NE gel on [C] IL-1β; [D] TNF-α and [E] stability studies.

The *in vitro* skin permeation study conducted using Franz diffusion cells revealed significant differences in drug delivery performance among the three formulations tested: free drug, NE, and NE gel [Fig. 10A]. The NE gel formulation showed higher permeation during the 12 h study period, with approximately 2-fold higher drug permeation than the free drug solution within the first 30 minutes. By 2 h, the NE gel had permeated 28% of the drug, a value significantly higher than that for the free drug solution. The NE formulation showed intermediate permeation, consistently delivering about 1.45 times more drug than the free solution. Remarkably, while the free drug solution demonstrated a plateau in permeation after 08 h, both NE formulations, specifically the NE gel, demonstrated sustained release. The results confirmed that Cp-NE enhanced drug permeation, and that the free drug and, further, the chitosan gel of Cp-NE showed sustained drug release, which may be attributed to controlled diffusion from the gel matrix.

### 3.8. Biocompatibility assessment

The biocompatibility of the formulations was evaluated using the MTT assay in HaCaT cells [Fig. 10B]. The results indicated that both Cp-NE and Cp-NE gel formulations were superior to the free drug in terms of their cell viability. At a concentration of 20 µg mL^−1^, the Cp-NE formulation showed a 1.02-fold increase in cell viability. In contrast, the Cp-NE gel showed a 1.04-fold improvement; at 80 µg mL^−1^, it provided 1.13-fold enhancement, and Cp-NE provided 1.09-fold enhancement in cell viability.

### 3.9. *In vitro* cytokine assay

The anti-inflammatory potential of free Cp, Cp-NE, and Cp-NE gel was estimated by the levels of pro-inflammatory cytokines IL-1β and TNF-α in LPS-stimulated RAW264.7 macrophages [Fig. 10C and D]. LPS stimulation significantly induced an inflammatory response in RAW264.7 macrophages, and the levels of IL-1β and TNF-α were increased 6.51- and 7.88-fold compared with the normal control group, respectively. This confirmed the successful induction of psoriasis-associated inflammatory conditions *in vitro*. Whereas the placebo NE gel showed cytokine levels comparable to the normal control, confirming that the excipients of the formulation did not provoke any inflammatory response. Cp-NE gel at 80 µg mL^−1^ decreased IL-1β from 78.46 pg mL^−1^ (LPS control) to 14.25 pg mL^−1^, a 5.51-fold decrease, whereas Cp-NE and free Cp decreased levels by 3.56- and 2.45-fold, respectively. Likewise, TNF-α levels decreased from 150.32 pg mL^−1^ in the LPS group to 31.62 pg mL^−1^ in the Cp-NE group, indicating 4.75-fold higher inhibition. On the other hand, both the Cp-NE gel and Cp showed 3.26- and 2.17-fold reductions, respectively. The results revealed that the Cp-NE gel exhibited significantly greater anti-inflammatory activity than free Cp. For instance, when using 40 µg mL^−1^, Cp-NE gel showed around 2.0 times lower IL-1β (22.09 *vs.* 44.26 pg mL^−1^) and TNF-α (48.04 *vs.* 94.13 pg mL^−1^) levels compared to free Cp. Also, when used alone, Cp-NE showed superior cytokine inhibition compared to free Cp, indicating the importance of nanoemulsion-based administration in improving Cp's intracellular anti-inflammatory activity. Importantly, treatment with Cp-NE gel at 80 µg mL^−1^ resulted in recovery of IL-1β and TNF-α levels that were almost similar to those in the normal condition, with levels only 1.18 and 1.66 times those in the normal condition. The results conclusively demonstrated that the use of Cp-NE-gel led to substantial enhancement of the anti-inflammatory efficacy of Cp against LPS-induced macrophage activation, which was much more effective than the free form of the drug, due to increased inhibition of IL-1β and TNF-α secretion in LPS-stimulated RAW264.7 macrophages. Moreover, the placebo NE gel showed negligible cytokine levels, revealing that the excipients did not induce the inflammatory markers. Thus, confirming that the improved suppression of IL-1β and TNF-α observed with Cp-NE and Cp-NE gel was due to the therapeutic effect of Cp rather than the formulation components.

### 3.10. Stability studies of optimized CME-NE formulation

The stability of the Cp-NEs was tested at 5 ± 3 °C and 25 ± 2 °C/60 ± 5% RH to assess storage impact. At 5 ± 3 °C, the formulation remained stable, with drug content decreasing slightly from 95.3% to 92.9% and globule size remaining nearly constant (122–125 nm), indicating minimal structural and chemical changes. Conversely, at 25 ± 2 °C/60 ± 5% RH, drug content dropped from 95.7% to 88.6%, and globule size increased from 122 nm to 145 nm, indicating instability due to globule aggregation or drug leakage (Fig. 10E). These findings demonstrate that NE formulation was comparatively more stable at refrigerated temperatures, where both drug content and physical integrity remained stable.

## 4. Conclusion

In the current study, optimization of the Cp-NE gel was accomplished successfully through a Box–Behnken design approach. The optimized formulation displayed satisfactory physicochemical characteristics like globule size of 122 nm, %EE of 89%, with satisfactory colloidal stability. Although the optimized formulation showed satisfactory physicochemical stability under the tested storage conditions, future studies will be performed for thermodynamic stability assessments and cloud point analysis in order to assess the stability of the formulation under various conditions. Formulation of the NE gel in a chitosan gel matrix enabled sustained drug release, improved skin permeability, improved cytocompatibility, and reduced pro-inflammatory cytokine levels relative to the free drug. In addition, the optimized formulation also showed good storage stability under refrigerated conditions. These findings highlight that the Cp-NE gel can be utilized as an effective topical formulation for the treatment of psoriasis. Further research is necessary to conduct thermodynamic stability testing as well as *in vivo* studies.

## Author contributions

Shiv Kumar Prajapati: data curation, investigation, writing – original draft, writing – review and editing, methodology, software, validation. Deepa Lashkari: data curation, investigation, writing – original draft, writing – review and editing, methodology. Sweta Acharya: writing – original draft, writing – review and editing. Meenakshi Bajpai: writing – original draft, writing – review and editing. Ankit Jain: project administration, supervision, writing – original draft, writing – review and editing.

## Conflicts of interest

There are no conflicts to declare.

## Abbreviations

BBD: Box–Behnken design
Cp: Clobetasol propionate
CQA: Critical quality attributes
DLS: Dynamic light scattering
EE: Entrapment efficiency
IPM: Isopropyl myristate
NE: Nano emulsion
PDI: Polydispersity index
QBD: Quality by design
QTPP: Quality target product profile
RPM: Rotation per minute
RSM: Response surface methodology
*S*_mix_
: Surfactant mix
ZP: Zeta potential

## Data Availability

The data supporting the findings are presented within the paper, and additional data may be requested from the authors.
